# App-based multimodal lifestyle-intervention for essential hypertension (HYPE): a decentralized randomised controlled trial

**DOI:** 10.3389/fdgth.2025.1672553

**Published:** 2025-10-15

**Authors:** Christian Beger, Marco Lehmann, Marisa Kaup, Lucy Jones, Ana Mijuskovic, Florian P. Limbourg

**Affiliations:** ^1^ Vascular Medicine Research, Department of Nephrology and Hypertension, Hannover Medical School, Hannover, Germany; ^2^ Oviva AG, Potsdam, Germany; ^3^ Oviva UK Limited, London, United Kingdom

**Keywords:** hypertension, lifestyle-intervention, digital therapeutics, randomised-controlled trial, App

## Abstract

**Background:**

Life-style interventions are effective in lowering blood pressure (BP) and reducing cardiovascular risk, but implementation is poor. We aimed to evaluate the efficacy of an app-based multimodal lifestyle intervention in reducing BP in patients with uncontrolled hypertension.

**Methods:**

In a decentralized, single-blinded, randomized-controlled trial, adults with uncontrolled hypertension by home BP measurement were randomized to 12 weeks of app-based multimodal lifestyle intervention or care as usual. The primary outcome was the difference of mean systolic BP after 12 weeks. Secondary outcomes were mean diastolic BP difference, and changes in body weight, health-related quality of life, and food literacy. An intention-to-treat analysis with multiple imputation by chained equations (MICE) was performed.

**Results:**

From June 13, 2024, to September 30, 2024, a total of 139 pts. were randomized with a mean baseline BP of 142/88 mmHg, of which 55% were females. After 12 weeks, there was a significantly lower systolic BP in the intervention group (−8.5 mmHg, [95% CI: −11.0 to −5.9], *p* < 0.001). At this time point, the intervention group also showed significantly lower diastolic BP (−5.06 mmHg), a larger relative reduction in body weight (−2.88%) as well as larger improvements in health-related quality of life and food literacy. Responder analysis confirmed that the effects were large and consistent across outcomes. No serious adverse events related to the intervention occurred during the trial.

**Conclusion:**

A digital multimodal lifestyle intervention may clinically improve general hypertension care and should be evaluated in larger trials.

**Clinical Trial Registration**: https://drks.de/search/de/trial/DRKS00034348, identifier (DRKS00034348).

## Introduction

Hypertension is the leading risk factor for disease and premature death, affecting a large proportion of the population worldwide (1, 2). In Germany, one in three individuals is diagnosed with the disease, while, at the population level, blood pressure (BP)-control is estimated at about 50%—contributing to an increasing burden of hypertension-mediated cardiovascular disease (3, 4). According to the World Health Organization early detection and effective treatment of hypertension are among the most cost-effective health interventions and should be prioritized by countries as part of their national health package delivered at primary care level (5). This requires effective implementation of cost-effective measures.

Lifestyle interventions, such as physical activity, a healthy diet, salt restriction or stress reduction are effective in lowering BP and reducing cardiovascular risk (6). Consequently, international guidelines recommend lifestyle intervention as the first-line treatment for hypertension (7–9). However, implementation in clinical practice is often limited by insufficient resources for structured patient education and guidance (10).

Digital health applications offer a promising approach to support patients in self-management by facilitating lifestyle changes, home-monitoring or improving adherence. However, the evidence for the efficacy of digital therapeutics is not unequivocal, especially with regard to digital lifestyle management (11–13). A recent meta-analysis of randomized-controlled trials (RCT) across various digital intervention techniques demonstrated significant reduction of BP independent of platform, but the effects were heterogeneous and the evidence level was considered low (14).

For smartphone apps, a meta-analysis of randomized trials demonstrated that apps are associated with a decrease in BP and an increase in medication adherence (13). In fact, a recent RCT conducted in hypertension centers demonstrated a positive effect of a lifestyle-intervention delivered by a digital therapeutic on BP in patients not receiving antihypertensive medication (12), but another trial with a digital therapeutic with focus on a multi-modal lifestyle intervention failed to show a significant effect on BP (11). Furthermore, first studies suggest that digital therapeutics combined with care as usual for hypertension can be a cost-effective treatment approach (15), which likely is influenced by specific healthcare system context.

We conducted a randomised, controlled, decentralized trial to evaluate the efficacy of an app-based multimodal lifestyle intervention in patients with uncontrolled hypertension under stable treatment. The digital therapeutic guides users in behavior modification in a structured fashion to achieve a healthy lifestyle. We assessed the potential impact on BP-management by analysing the effect on systolic BP (SBP), body weight, quality of life and nutrition related health literacy.

## Materials and methods

### Study design

The HYPE study was a phase III, randomised, controlled, single-blinded clinical trial conducted in Germany, aiming to investigate the effect of an app-based, multimodal intervention (“Oviva Direkt Hypertension”) on BP in patients with hypertension. The trial design was a two-arm, parallel-group comparison of Oviva Direkt Hypertension plus care as usual vs. care as usual alone. The study was fully decentralised, with all participant interactions conducted remotely via telephone or video calls. Ethics Committee approval was provided on 28th May 2024 by the Ethics Committee of the Medical Association of Lower Saxony, Germany with study ID HYPE001. The trial is registered in the German Clinical Trial Register under the identifier DRKS00034348 (registration date: 10 June 2024; https://drks.de/search/de/trial/DRKS00034348). There were no important changes to methods after trial commencement. After collection of the primary outcome measures, participants were asked to provide feedback about the study.

### Participants

Participants were recruited nationwide through a digital recruitment campaign and from physician referrals. Potential participants completed an online pre-screening questionnaire. Those meeting initial eligibility criteria provided home-BP-measurements over seven days (minimum of three consecutive days). Individuals with a mean SBP >135 mmHg were scheduled for a remote screening visit with a study physician. The study physician enrolled eligible and consenting patients for the study. Adults aged 18–75 years with essential hypertension (mean home SBP >135 mmHg) and stable antihypertensive treatment with fewer than four antihypertensive drugs for at least three months were eligible for the study. Among others, participants were required to have sufficient German language skills, a compatible smartphone and a validated BP-monitor. Key exclusion criteria included a systolic BP ≥180 mmHg, recent cardiovascular events (within six months), severe cognitive impairment and weight loss >5% in the past six months. The complete inclusion and exclusion criteria are provided in the Supplementary Material S1. All participants provided informed consent.

### Randomisation and masking

Eligible participants were randomly assigned (1:1) to either the intervention or control group using block randomisation (block sizes of 4, 6, and 8, without stratification). An external statistician, not otherwise involved in the study, generated the concealed allocation sequence, which was implemented via the Sealed Envelope platform (http://www.sealedenvelope.com). The study team used Sealed Envelope to obtain each participant's allocation after enrolling their identifier, ensuring concealment from staff involved in recruitment and data collection. Due to the nature of the intervention, participants were aware of their group assignment. However, study visits and outcome assessment were performed in a blinded fashion by the study team. Data were recorded with a certified electronic data capture system (Open Clinica, http://www.openclinica.com), which concealed group assignment of participants during study visits. Study visits were not used for data collection but for support in procedural questions and safety checks.

### Procedures

The intervention group received access to Oviva Direkt Hypertension, a CE-marked, app-based multimodal lifestyle intervention, for 12 weeks in addition to care as usual. Oviva Direkt Hypertension focuses on self-monitoring, self-management, and education, delivered through the Oviva Direkt smartphone app. Patients installed this digital therapeutic on their own mobile phone. The intervention included guidance on body weight reduction, a low-carbohydrate and low-salt diet (based on DASH diet principles), increased physical activity, stress management, reduction in alcohol consumption, and meal plans. The intervention was self-guided, with participants following in-app instructions. Further details are provided in Supplementary Figure S2. Self-monitoring was enabled through tracking of BP, weight, meals, drinks, and physical activity. Self-management was supported through goal setting, feedback, and reminders via in-app alerts and push notifications. Educational content covered hypertension management, including the lifestyle modifications. Participants received a support call from an Oviva contact person within the first two weeks to ensure proper use and safety. Ongoing support was available via in-app chat or telephone.

The control group received care as usual for hypertension, consisting of usual treatment within the German healthcare system (primarily antihypertensive medication) and an educational brochure from the Deutsche Hochdruckliga outlining lifestyle recommendations. They received access to the digital therapeutic after the 12-week data collection period, but this usage was not part of the study.

Data collection used OpenClinica as a certified electronic data capture system with fully automated scheduling of study visits (http://www.openclinica.com). Data collection for BP, weight and questionnaires (SF-8, SFLQ) was performed at baseline, week 4, week 8 and week 12 via remote self-reported electronic patient reported outcomes (ePRO). Body mass index (BMI) was calculated from self-reported height and weight. Data on app usage (intervention compliance) was automatically collected for the intervention group via the smartphone app. There were no changes to trial outcome measures after the trial commenced. Medication changes were recorded at all follow-up study visits.

Adherence to the app intervention was defined as at least one app activity per week. The digital lifestyle intervention does not operate with a strict coaching/checking schedule, but instead with a motivational concept based on personal schedules and preferences. The measure of one app activity per week represents a minimum criterion that patients remain onboard throughout the intervention period.

### Outcomes

The primary outcome was mean SBP at week 12, measured by home blood pressure monitoring (HBPM) according to current guideline recommendations (7, 8). Participants were instructed in appropriate BP-measurement (Supplementary Content S3) and BP was recorded for seven consecutive days (duplicate measurements morning and evening; minimum of three consecutive days). Secondary outcomes included percent change in body weight from baseline to week 12, mean diastolic BP at week 12, change in health-related quality of life from baseline to week 12 (assessed via the SF-8 questionnaire) and change in food-literacy from baseline to week 12 (assessed via the SFLQ questionnaire). A complete description of the outcomes is provided in Supplementary Material S4. Adverse events (AEs) and serious adverse events (SAEs) were collected, recorded and reported according to the sponsor's Standard Operating Procedure (SOP), aligned with ICH guidelines and local regulatory requirements. AEs and SAEs were summarised overall, by severity and by relationship to the medical device. Causality assessment was performed, classifying events as not related, possible, probable, or causal relationship.

### Statistical analysis

A total of 134 participants were planned to be enrolled, accounting for an expected 25% dropout rate, resulting in 100 completed cases. This sample size provided 90% power to detect a medium effect size (*d* = 0.65) on the primary outcome (systolic blood pressure at 12 weeks, analyzed via ANCOVA as described in this section below), with a two-sided alpha level of 0.05. No adjustment for multiple testing was required, as the primary success criterion was based on a single statistical test.

The primary statistical method was an intention-to-treat (ITT) analysis of covariance (ANCOVA). The primary outcome (mean SBP at week 12) was compared between groups, adjusting for baseline SBP, age, gender, baseline weight, and changes in antihypertensive medication. Adjusted group differences will be reported. Missing data were handled using multiple imputation by chained equations (MICE). Secondary outcomes were analyzed similarly, with ANCOVA models adjusting for baseline values of the respective outcome, age, and gender. Subgroup analyses were planned to explore differential effects by age group (<60 years, ≥60 years) and gender. Sensitivity analyses were planned to assess the robustness of the primary analysis, including different imputation methods and a per-protocol (PP) analysis. Safety analyses were descriptive, comparing the number of reported adverse events between groups. All statistical analyses were performed by an external statistician according to a prespecified statistical analysis plan, using R statistical software. No interim analyses were conducted.

### Role of funding source

Planning, implementation and analysis of the study was financed by Oviva AG, Potsdam, Germany. The interpretation and analysis of the data as well as the writing and editing of this article received institutional support from Hannover Medical School, Germany.

## Results

**Figure 1:**
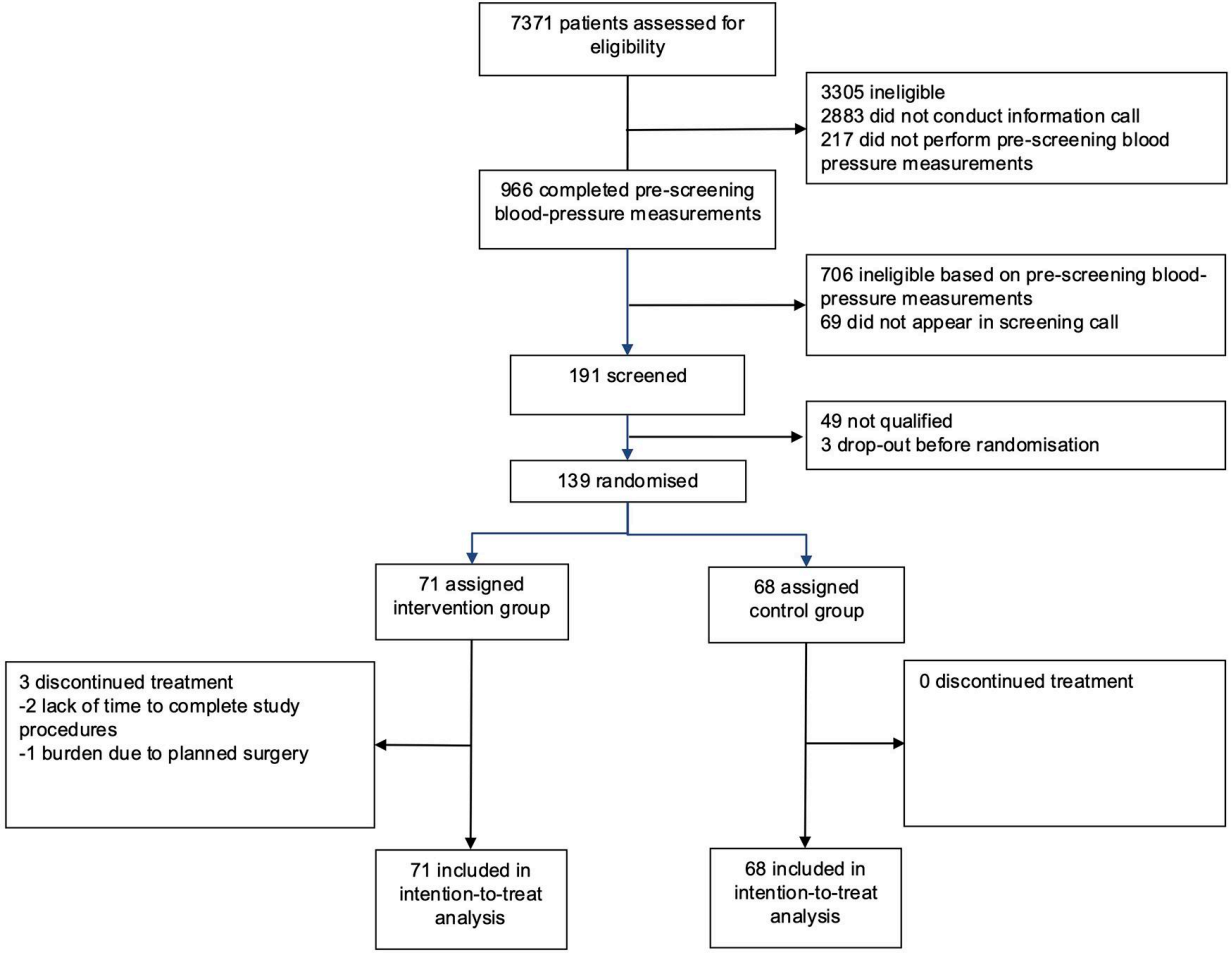
Study participant CONSORT flowchart.

Participants were recruited from June 13, 2024 to September 30, 2024 and data collection was finalized on December 30, 2024. 7371 individuals completed the online pre-screening questionnaire. After pre-selection and an initial information call, 966 (13%) participants provided home BP values from 7 consecutive measurement days. A total of 191 (2.6%) participants with uncontrolled hypertension were further assessed for eligibility by video interview, from which a total of 139 (1.9%) participants were finally randomised: 71 were allocated to the intervention group and 68 were assigned to the control group (Figure 1).

The mean age (SD) of the participants was 54.6 (9.9) years, and 77 (55%) were female. The mean SBP (SD) was in the uncontrolled range and comparable for both groups [Intervention 141.9 (4.2) mmHg, control 141.8 (5.2) mmHg]. At baseline, the majority of participants received antihypertensive monotherapy, with 37 (54%) in the control and 29 (41%) individuals in the intervention group. One participant with four classes of antihypertensive medication was accidentally randomised. The patient remained in the intention-to-treat analysis but was excluded from the per-protocol analysis. The mean BMI (SD) at baseline was 33.0 (6.7) kg/m^2^ in the intervention group and 33.1 (6.0) kg/m^2^ in the control group. Intervention and control groups were comparable in terms of baseline characteristics (Tables 1, 2).

**Table 1:** Baseline characteristics of study participants.

Characteristics	Intervention, (*n* = 71)	Control, (*n* = 68)
Age [Mean years (SD)]	56.1 (8.9)	53.0 (10.7)
Gender		
Female	39 (55%)	38 (56%)
Male	32 (45%)	30 (44%)
BMI [Mean kg/m² (SD)]	33.0 (6.7)	33.1 (6.0)
Number of Antihypertensive Medications		
1	29 (41%)	37 (54%)
2	31 (44%)	18 (26%)
3	11 (15%)	12 (18%)
4	0 (0%)	1 (1.5%)

Mean (SD); *n* (%).

**Table 2:** Primary and secondary outcome results.

Outcome	Intervention (Mean, SD)			Control (Mean, SD)			Adjusted mean difference (SE)	*t* (*df*)	*p*	*d*	95% CI
Primary outcome	Baseline	12 weeks	Change	Baseline	12 weeks	Change					
Systolic blood pressure (mmHg)	141.9 (4.2)	130.7 (9.2)	−11.17 (9.23)	141.8 (5.2)	139.6 (6.4)	−2.46 (6.22)	−8.45 (1.30)	−6.49 (132)	<0.001	−0.94	−1.24 to −0.64
Secondary outcomes											
Diastolic blood pressure (mmHg)	87.5 (5.0)	80.1 (7.8)	−6.93 (5.85)	88.8 (6.2)	86.7 (6.9)	−1.6 (4.38)	−5.06 (0.95)	−5.32 (132)	<0.001	−0.85	−1.18 to −0.52
Weight change (%)	99.2 (19.5)	95.4 (19.0)	−3.65 (3.19)	98.9 (18.0)	98.4 (18.6)	−0.5 (2.96)	−2.88 (0.56)	−5.16 (133)	<0.001	−0.89	−1.25 to −0.54
Health related quality of life change (SF-8)	39.7 (11.4)	46.5 (10.5)	6.7 (10.1)	39.3 (10.7)	40.6 (10.4)	1.4 (7.9)	5.69 (1.45)	3.92 (132)	<0.001	0.73	0.35–1.10
Food literacy change (SFLQ)	34.8 (5.9)	41.7 (4.5)	6.8 (5.8)	34.7 (5.3)	37.0 (4.8)	1.9 (3.5)	4.74 (0.72)	6.62 (132)	<0.001	1.33	0.91–1.75

Descriptives for baseline, 12 weeks, and change use observed data only. Adjusted mean difference and statistical test results are based on MICE imputed data and covariate adjustment.

**Figure 2:**
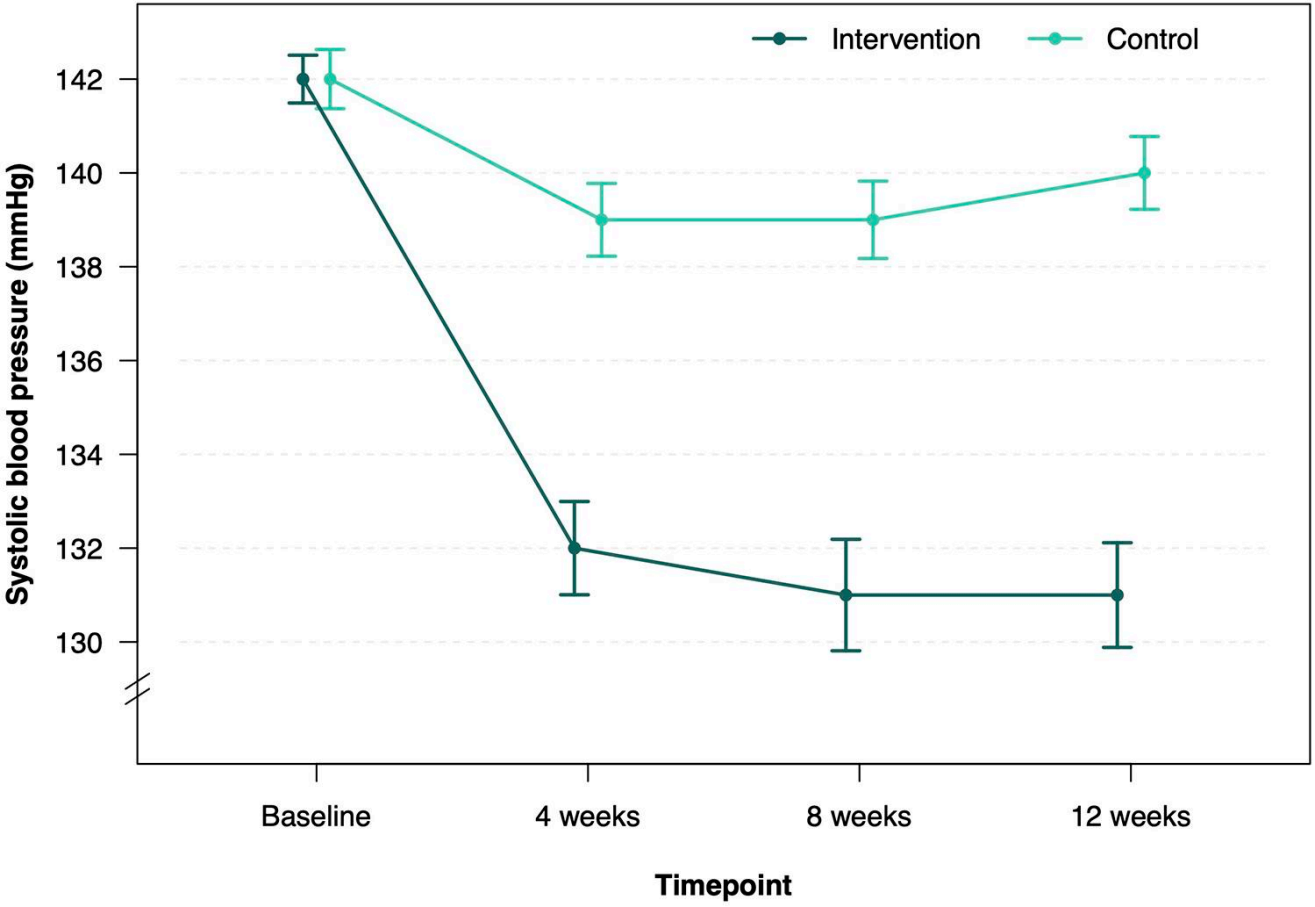
Systolic BP time course. Mean SBP (SEM) per group at indicated follow-up (wks).

Twelve-week follow-up was completed in 68 patients in the intervention group (95.8%) and 68 patients (100%) in the control group (Figure 1). After 12 weeks, the mean SBP decreased by 11.2 mmHg–130.7 mmHg in the intervention group and by 2.5 mmHg–139.6 mmHg in the control group (Table 2, Figure 2). Consistent with previous reports, significant BP effects were apparent after 4 weeks (12). The primary endpoint, the ANCOVA adjusted between-group-difference at 12 weeks, was −8.45 mmHg (95% CI, −11.0 mmHg to −5.9 mmHg, *P* < 0.001, *d* = −0.94; Table 2). In a further sensitivity analysis, the results were consistent using the per-protocol population (between group difference −8.19 mmHg, 95% CI, −11.0 mmHg to −5.4 mmHg, *P* < 0.001, *d* = −0.91, Supplementary Table S5). In addition, 75.8% of all participants in the intervention group experienced a decrease in SBP of at least 5 mmHg. In the control group, this proportion was 35.9% (Supplementary Table S6). A logistic regression model confirmed that the intervention group was more than five times more likely to achieve a 5 mmHg improvement in SBP than the control group (OR = 5.78, 95% CI, 2.67–13.2). Subgroup analyses of the ITT sample revealed consistency of the intervention effect within the subgroup of patients under 65 years of age and within the male and female subgroups (Supplementary Tables S7–S9). Due to small sample size of patients over 65 years, the observed intervention effect was not statistically significant in this subgroup.

In secondary endpoint analysis, the mean diastolic BP (SD) decreased by 6.9 mmHg to 80.1 (7.8) mmHg in the intervention group and by 1.6 mmHg in the control group to 86.7 (6.9). The ANCOVA adjusted between-group-difference at 12 weeks was −5 mmHg (95% CI, −6.9 mmHg to −3.2 mmHg, *d* = −0.85, *P* < 0.001). In addition, the ANCOVA adjusted mean difference in percent weight change between intervention and control group was −2.88% of baseline body weight (95% CI, −4.0% to −1.8%, *P* < 0.001, *d* = −0.89), Table 2). 57.6% of the participants in the intervention group lost >3% of initial body weight, while only 10.6% in the control group lost >3%. The SF-8 health-related quality of life (HRQoL) increased by 6.7 (10.1) in the intervention group and by 1.4 (7.9) in the control group, resulting in an ANCOVA adjusted between-group-difference of 5.69 points (95% CI, 2.8–8.6, *P* < 0.001, *d* = 0.73, Table 2). Significant improvements were observed in the intervention group across all domains and items (Supplementary Table S10). In addition, food literacy also improved significantly more in the intervention group [6.8 (5.8)] compared to the control group [1.9 (3.5)], with an ANCOVA adjusted between-group-difference of 4.74 (95% CI, 3.3–6.2, *P* < 0.001, *d* = 1.33; Table 2). Subgroup analysis of the ITT sample regarding secondary outcomes revealed consistency of the intervention effect (Supplementary Tables S7–S9). In the intervention group, patients under 65 years of age as well as males and female subgroups showed significantly higher weight loss, lower diastolic blood pressure, improved quality of life, and improved food literacy, while patients over 65 years showed significantly higher weight loss.

**Figure 3:**
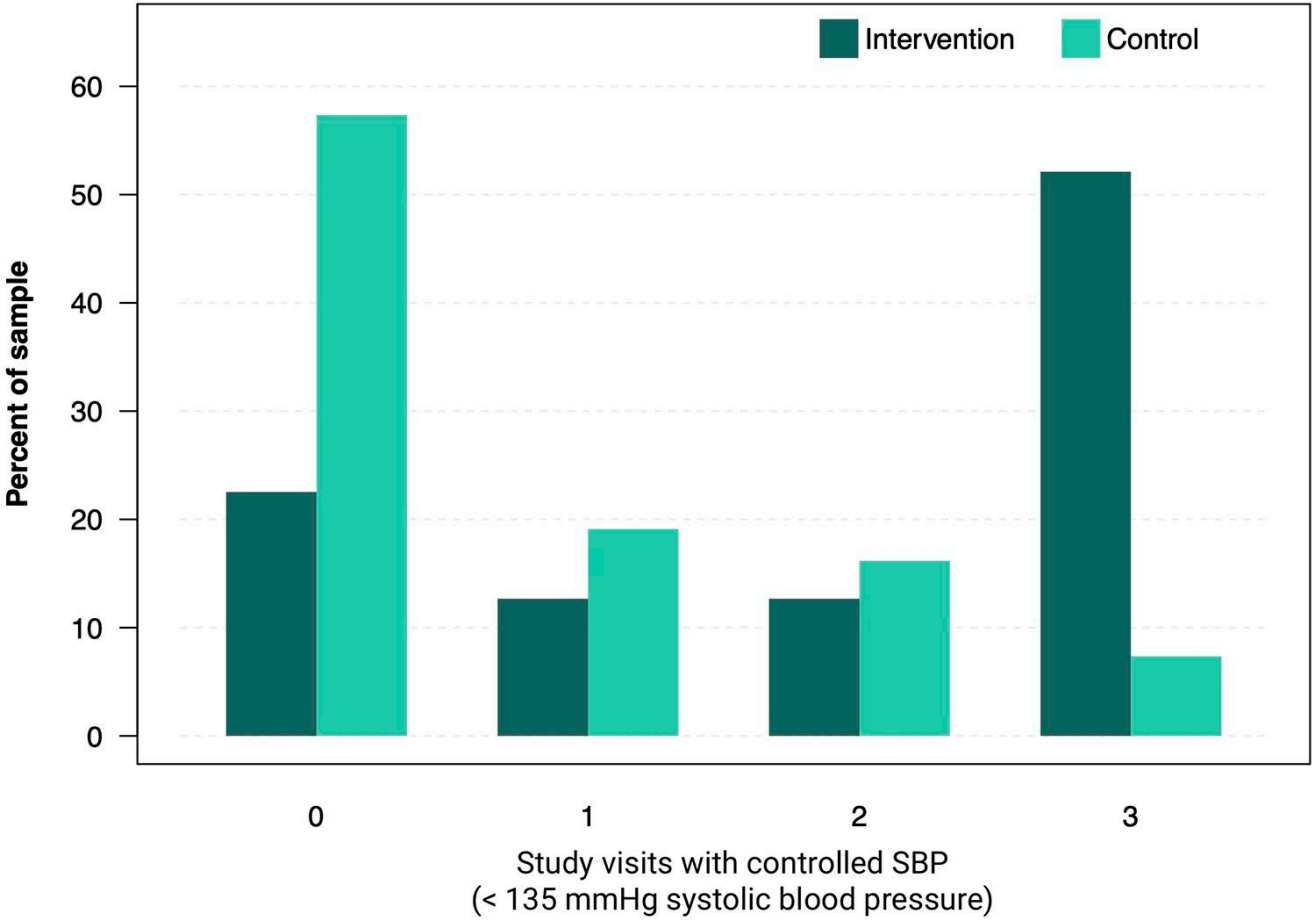
Number of study visits recorded with controlled systolic BP. Bars show the proportions of patients with average SBP < 135 mmHg in control and intervention group.

Extended analysis revealed that SBP control (SBP < 135 mmHg) at week 12 was achieved by 47 (69%) patients in the intervention group and 16 (24%) patients in the control group. Time-in-range analysis of SBP, conducted as an exploratory endpoint, revealed that 52% of patients in the intervention group presented with controlled SBP at all three study visits, vs. 7.4% in the control group. Conversely, 22.5% of patients in the intervention group never presented with controlled SPB during study visits, compared to 57.4% in the control group (*χ*^2^ = 34.9, *df* = 3, *P* < 0.001, Figure 3). Adherence to the app intervention, defined as engaging in at least one app activity per week, was 97.2% after 12 weeks with consistent high adherence rates throughout the study period (Supplementary Table S11). No adverse events related to the medical device or study conduct were observed in either the intervention or control group. The trial is reported according to Consort 2025 format (Supplementary Table S12) with a study synopsis (Supplementary Table S13).

## Discussion

In this decentralised, randomised controlled trial of patients with uncontrolled hypertension, a digital therapeutic for multimodal lifestyle intervention significantly reduced SBP and improved BP control after 12 weeks compared to usual care. The intervention also significantly reduced diastolic BP, promoted weight loss, and improved quality of life and food literacy.

This study demonstrates that a digitally enabled lifestyle intervention without physician support can lead to clinically meaningful improvements in BP-control. The intervention group showed a significantly lower mean SBP of approximately 8.5 mmHg relative to the control group—findings that were robust across both the intention-to-treat and per-protocol analyses and were supported by the outcomes for diastolic BP, BP control, and time-in-range analysis. There is a linear relationship between the level of SBP reduction and the risk reduction for serious cardiovascular events and all-cause mortality. Based on results from an meta-analysis, the SBP reduction observed with this intervention may be associated with an absolute risk reduction of more than 20% for cardiovascular events and around 13% for all-cause mortality, if delivered over a longer period in a sustained manner (1). The observed BP reduction in the current trial falls within the upper range of published data (11, 12, 16) and is generally comparable to the effects achieved with pharmacological monotherapy (17) or interventional approaches (18). However, due to the short study duration and relatively small population, this serves only as an approximate reference for the effect size- a direct comparison of these therapeutic approaches is not possible.

The effects of the app intervention extended beyond BP-control. Participants in the intervention group experienced a significant weight reduction—with 57.6% losing more than 3% of their baseline body weight compared to 10.6% among controls. This is comparable to similar weight improvements with a related digital therapeutic in a previous study in obese patients (19). In addition, notable enhancements were observed in health-related quality of life (an adjusted between-group difference of 5.69 points on the SF-8 scale) and improvements in food literacy. These improvements suggest that the intervention catalyzed behavioral changes that are likely to sustain long-term cardiovascular benefits. The high adherence to the digital platform indicates that such interventions are both feasible and acceptable to patients. The ease of use and consistent interaction with the app likely facilitated self-monitoring, reinforced healthy behaviors, and provided timely feedback—factors which are essential for sustaining behavioral change and optimizing clinical outcomes in hypertension management.

Furthermore, in subgroup analysis, the intervention showed consistent effects on SBP control and secondary endpoints in males and females and patients under the age of 65 years, indicating effectiveness of the intervention across genders and younger patients. For patients of a higher age, results remain inconclusive because of the small sample size available. This should be clarified in a larger trial.

Our study's findings build on the growing body of evidence supporting digital health interventions for hypertension management. The Smart Hypertension Control Study aimed to investigate the effect of a smartphone coaching app compared to a BP-tracking app on SBP. After six months, no significant difference was observed between the control and intervention group. In contrast to the current study, the control group only received information on self-monitoring but not on lifestyle management (11). The HERB-DH1 trial specifically evaluated the effect of a digital lifestyle intervention vs. conventional lifestyle management. After 12 weeks, the use of a digital therapeutic resulted in a between-group difference of −4.3 mmHg in home BP compared to conventional lifestyle management. The HERB-DH1 only included hypertensive patients without medication and trial visits were managed in hypertension centers (12). In contrast, our study demonstrates a significant BP-reduction in a decentralized trial design and stable antihypertensive-medication throughout the follow-up. In a recent trial with a comparable design involving 102 participants, the digital lifestyle intervention resulted in a significant −5.06 mmHg between-group difference in SBP. However, no differences were observed regarding diastolic BP (16). Furthermore, in a recent systematic review and meta-analysis of RCTs across various digital intervention techniques, digital interventions yielded a pooled reduction in systolic blood pressure of −3.6 mm Hg (95% CI −5.2 to −2.0) and diastolic blood pressure of −2.6 mm Hg (–3.8 mmHg to −1.1 mmHg). Although the effects were consistent across platforms, the various digital therapeutics differed in both technology and content (14), which may have important clinical effects. For example, a recent meta-analysis of self-monitoring trials revealed that the degree of BP reduction depends on the intensity of intervention and particularly its integration into the medical treatment process. The most pronounced BP-reduction (−6.1 mmHg, 95% CI: −9.0 mmHg to −3.2 mmHg) was observed in trials where participants received intensive, continuous personal support throughout the study. In contrast, self-monitoring in itself was associated with only a slight reduction in BP (20), which could explain the modest BP-reduction observed in the control group of the current trial in addition to effects of conventional lifestyle management. Consistent with this, standalone apps such as in the Smart Hypertension Control Study often failed to achieve significant BP-reduction despite a multi-modal approach (11). In contrast, the HERB-DH1 system, which consisted of a smartphone app and a web application, enabled direct interaction between users and physicians (12). Another study which demonstrated a significant BP-reduction analysed an app which offered not only lifestyle coaching but also enabled remote access to physician consultations (21). In the digital therapeutic currently analysed, no interaction with the treating physician was implemented.

International guidelines recommend lifestyle interventions as the first-line treatment for hypertension (7–9). However, their implementation in clinical practice is often challenging (8, 22, 23). Barriers include insufficient patient knowledge, as well as constraints in healthcare professionals' ability to effectively convey relevant information and support behavioural changes (23). A recent survey revealed that only 40%–50% of patients felt sufficiently informed about potential lifestyle interventions (24). Digital therapeutics focusing on lifestyle management seem to have the potential to lower BP and improve BP-control (12, 25). Compared to the control group, food literacy significantly improved throughout our study, indicating effective educational support. To promote behavioral changes, strategies such as goal setting and monitoring are recommended, both of which are implemented in this digital therapeutic. The fact that antihypertensive medication in our study remained stable during follow-up is consistent with app-usage induced BP reduction mediated by lifestyle effects. Multiple lifestyle-strategies such as physical activity, healthy diet or stress reduction are implemented in the digital therapeutic. Previous studies have shown that single interventions (e.g., DASH diet, aerobic exercise) as well as comprehensive lifestyle-interventions are effective in reducing BP (6, 22). However, data on digital lifestyle interventions are still limited (14). In addition to exercise and a healthy diet, weight reduction can have a significant impact on BP-values and control. For example, a meta-analysis of 25 RCTs demonstrated a BP-reduction of 1 mmHg for every 1 kg of body weight lost (26). In this study, participants in the intervention group lost 2.88% more body weight compared to the control group.

Limitations of this study include lacking information on office BP values. However, participants performed structured, guideline-compliant HBPM, which has significantly higher prognostic relevance than office BP values and is currently recommended by guidelines for the management of hypertension (7, 8). Thus, HBPM appears to be a suitable method for evaluating the effect of a digital therapeutic for self-management. However, standardization remains a challenge compared to office BP measurements. Participants were instructed on correct BP-measurement and were required to use a validated device. Furthermore, due to the nature of the intervention, blinding of patients was not possible and restricted to study staff and the analytics team. While endpoint data capture was performed remotely and independently by patients via the database system, there remains an inherent potential for bias in this type of study design.

Although the inclusion criteria were broad, the findings are not fully generalizable. Recruitment required a smartphone and a validated BP monitor, which may limit participation of certain groups—such as frail or care-dependent individuals with limited digital access and potentially certain socioeconomically disadvantaged patients. Furthermore, due to specific coaching concepts and contents of individual apps, the findings of this study cannot be generally transferred to other digital therapeutics. The study demonstrated effects over a 12-week period, which is a commonly chosen duration in comparable studies (12, 16). Currently, no conclusions can be drawn regarding the sustainability of the effects. A longer follow-up study over six months is planned, while real-world clinical data could provide additional insights.

## Data Availability

The original contributions presented in the study are included in the article/Supplementary Material, further inquiries can be directed to the corresponding author.
