# Supplementary material for: App-based multimodal lifestyle-intervention for essential hypertension (HYPE): a decentralized randomised controlled trial
Source: Front Digit Health. 2025 Oct 15;7:1672553. doi: 10.3389/fdgth.2025.1672553 (PMC12569480; doi:10.3389/fdgth.2025.1672553)
Supplement: Supplementary file 1 [file Datasheet1.pdf]

Supplementary Material

App-Based Multimodal Lifestyle-Intervention for Essential Hypertension (HYPE): A Decentralized Randomised Controlled Trial

S1. Inclusion and Exclusion criteria

Inclusion criteria

- Adults aged 18-75 years
- Fulfilling the criteria for essential hypertension, which is an average systolic blood pressure > 135 mmHg measured at rest with validated devices and according to the 2023 ESH Guidelines for the management of arterial hypertension[1] for home-blood pressure monitoring.
- Stable antihypertensive treatment for at least three months
- Concurrent use of fewer than 4 antihypertensive drugs
- Treatment goal to reduce systolic blood pressure using a lifestyle intervention
- Valid and working email address, telephone number, and internet access
- Sufficient skill in the German language for participation
- Signed informed consent
- Smartphone available and compatible with the Oviva Direkt app
- Possession of a validated blood pressure monitor
- Motivation and ability to make lifestyle changes

Exclusion criteria

- Average systolic blood pressure at rest ≥ 180 mmHg
- Patient safety reasons (e.g., breastfeeding, pregnancy, concurrent insulin treatment)
- Cardiovascular event in the last 6 months (e.g., stroke, heart attack, cerebral haemorrhage)
- Severe cognitive and intellectual impairment
- Current or recent participation (within the past 12 months) in a digital lifestyle intervention or clinical investigation targeting blood pressure and/or body weight reduction
- Weight loss >5% in the past 6 months (intentional or unintentional)
- Living in a household with another study participant for a digital lifestyle intervention
- Terminal diagnosis with a life expectancy of less than 24 months
- Planned long-term absence > 3 weeks

S2. Oviva Direkt app screenshots showing different ways to use the app.

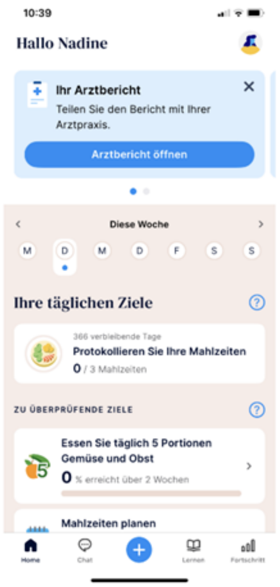

1. App Home Screen

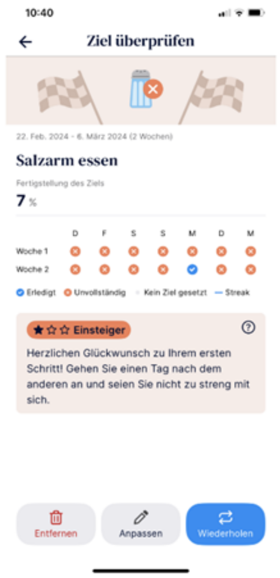

2. Goal setting in the app for self management

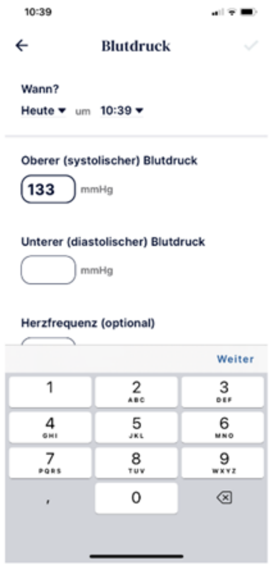

3. Self-monitoring of blood pressure in the app

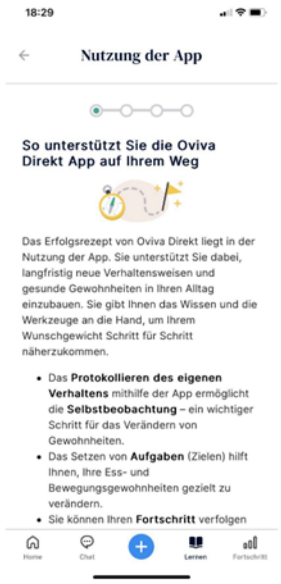

4. Learning content in the app

S3. Instructions for home blood pressure measurement for study participants (English translation).

How to measure your blood pressure

Automatic blood pressure monitors are suitable for measuring your blood pressure yourself. In addition to these guidelines, please also follow the measuring instructions for the blood pressure monitor. Please measure your blood pressure on 7 consecutive days according to the instructions in this document and enter the values in the questionnaire that you have received by e-mail for this purpose. We will also provide you with a blood pressure diary in which you can record your measurements.

1. Time of measurement

- Please measure your blood pressure on seven consecutive days.
- Take 2 consecutive measurements in the morning and evening, 1-2 minutes apart.
- Morning means before the day begins (before coffee); evening means when the day is finished, before going to bed.
- If you work shifts, measure immediately after sleeping and before going to bed.
- It is advisable that the measurement times are the same every day and that the general conditions of the measurements are as similar as possible so that the values can be compared with each other.
- You should only take your antihypertensive medication after the measurement.
- We advise you to take several measurements a day, as this will give you a much more accurate picture of your actual blood pressure than a single measurement. Individual measurements are strongly influenced by what you have done before or how you are currently feeling.

2. Preparations

- Your bladder should be empty.
- Try to avoid physical and emotional stress 30 minutes before the measurement. Smoking, coffee, food and sport in the 30 minutes before the measurement can also affect the results.
- Sit quietly for at least 3-5 minutes before the measurement (resting also means not talking on the phone, watching TV or typing messages).
- Put on the cuff according to the device's measurement instructions.

3. During the measurement

- Carry out the measurement in accordance with the device's measurement instructions.
- Sit upright and place your legs loosely next to each other.
- Place your left forearm comfortably and relaxed on a flat table so that the cuff you are wearing on your arm is at heart level.
- Try not to speak during the measurement (until the cuff is fully deflated)

S4. Trial Outcomes

Primary outcome

Mean systolic blood pressure measured by HBPM at week 12

Baseline blood pressure was measured for seven consecutive days, adhering to guidelines[1,2]. Follow-up measurements were conducted at 4, 8, and 12 weeks, each over seven consecutive days within a ±7 day window of the scheduled check-in (28±7, 56±7, 84±7 days). If a patient provided at least three consecutive days of measurements within the window, all valid measurements (excluding outliers) were included.

Blood pressure was measured both morning and evening. Two systolic and diastolic readings, two minutes apart, were recorded immediately upon waking and before sleep.

Secondary outcomes

1. Mean of percent body weight change between baseline and 12 weeks measured by HBPM
   Body weight is aggregated at baseline, 4, 8, and 12 weeks. A minimum of two measurements per timepoint were deemed acceptable for outcome calculation.

2. Mean diastolic blood pressure measured by HBPM at week 12
   (see systolic blood pressure measurements)

3. Mean of health-related quality of life change between baseline and 12 weeks measured by SF-8 questionnaire score
   As an overall indicator of quality of life the total sum score is calculated as previously published[3].

4. Mean of patient food literacy between baseline and 12 weeks measured by SFLQ questionnaire score
   The twelve items of the Short Food Literacy Questionnaire (SFLQ) were summed to a total score as previously published[4]. Because item 2 is a composite of five ratings, these ratings were averaged first and then entered as a response to item 2 in the summation, as recommended in the source.

Table S5. Primary and secondary outcome results - per protocol analysis.

	Intervention (Mean(SD))			Control (Mean (SD))			Adjusted mean difference (SE)	*t (df)*	*p*	*d*	95% CI
Primary outcome	Baseline	12 weeks	Change	Baseline	12 weeks	Change					
Systolic blood pressure (mmHg)	141·9 (4·2)	130·7 (9·2)	-11·17 (9·23)	141·8 (5·2)	139.6 (6.4)	-2·46 (6·22)	-8.19 (1.40)	-5.85 (114)	< 0.001	-0.91	-1.23 - -0.59
Secondary outcomes											
Diastolic blood pressure (mmHg)	87·5 (5·0)	80·1 (7·8)	-6·93 (5·85)	88·8 (6·2)	86.7 (6.9)	-1·6 (4·38)	-5.46 (0.97)	-5.63 (110)	< 0.001	-0.92	-1.25 - -0.58
Weight change (%)	99·2 (19·5)	95·4 (19·0)	-3.65 (3.19)	98·9 (18·0)	98·4 (18·6)	-0.5 (2.96)	-3.00 (0.55)	-5.43 (114)	< 0.001	-0.93	-1.29 - -0.58
Health related quality of life change (SF-8)	39·7 (11·4)	46·5 (10·5)	6.7 (10.1)	39·3 (10·7)	40·6 (10·4)	1.4 (7.9)	6.47 (1.40)	4.63 (117)	< 0.001	0.83	0.46 - 1.19
Food literacy change (SFLQ)	34·8 (5·9)	41·7 (4·5)	6.8 (5.8)	34·7 (5·3)	37·0 (4·8)	1.9 (3.5)	4.60 (0.69)	6.65 (114)	< 0.001	1.29	0.88 - 1.69

Descriptives for baseline, 12 weeks, and change use observed data only. Adjusted mean difference and statistical test results are based on covariate adjustment.

Table S6. Systolic blood pressure response at 12 weeks.

	Intervention n = 71	Control n = 68
>= 5 mmHg reduction	50 (75.8%)	23 (35.9%)
0-4.9 mmHg reduction	12 (18.2%)	32 (50.0%)
BP increase	4 (6.1%)	9 (14.1%)

n (%)

Table S7. Descriptive statistics of outcomes by age group, ITT population, observed values

	Age Group			
	**> 65**		**≤ 65**	
**Characteristic**	**Intervention**, n = 12[1]	**Control**, n = 6[1]	**Intervention**, n = 59[1]	**Control**,nN = 62[1]
Gender				
Female	7 (58.3%)	3 (50.0%)	32 (54.2%)	35 (56.5%)
Male	5 (41.7%)	3 (50.0%)	27 (45.8%)	27 (43.5%)
Systolic Blood Pressure (mmHg)	130.2 (8.1)	137.1 (5.7)	130.7 (9.4)	139.8 (6.4)
Weight change (%)	-4.1 (3.2)	1.2 (5.8)	-3.6 (3.2)	-0.7 (2.5)
Diastolic Blood Pressure (mmHg)	75.5 (8.7)	84.2 (5.1)	81.0 (7.4)	86.9 (7.1)
Quality-of-Life (SF-8) change	4.1 (7.0)	-1.5 (5.7)	7.3 (10.4)	1.5 (7.9)
Food Literacy (SFLQ) change	7.0 (7.8)	3.0 (2.2)	6.7 (5.4)	1.8 (3.6)

[1]n (%); Mean (SD)

Table S8. Descriptive statistics of outcomes by gender, ITT population, observed values

	Gender			
	**female**		**male**	
**Characteristic**	**Intervention**, n = 39[1]	**Control**, n = 38[1]	**Intervention**, n = 32[1]	**Control**, n = 30[1]
Age	56.5 (7.9)	53.6 (10.6)	55.7 (10.2)	52.2 (10.9)
Age Group				
> 65	7 (17.9%)	3 (7.9%)	5 (15.6%)	3 (10.0%)
≤ 65	32 (82.1%)	35 (92.1%)	27 (84.4%)	27 (90.0%)
Systolic Blood Pressure (mmHg)	129.5 (9.2)	138.9 (6.0)	132.2 (9.1)	140.4 (6.9)
Weight change (%)	-4.4 (3.4)	-1.0 (1.8)	-2.8 (2.7)	0.2 (4.0)
Diastolic Blood Pressure (mmHg)	79.3 (7.9)	86.4 (6.2)	81.2 (7.7)	87.0 (7.9)
Quality-of-Life (SF-8) change	7.8 (10.8)	3.2 (7.2)	5.5 (9.0)	-1.3 (7.8)
Food Literacy (SFLQ) change	7.5 (6.3)	1.4 (3.3)	5.8 (5.0)	2.6 (3.7)

[1]Mean (SD); n (%)

Table S9. Subgroup effects for primary and secondary endpoints, ITT population, MICE-imputed values.

						95% CI						
	**Stratum**	**Group**	**EMM**	**SE[1]**	**DF[2]**	**lower**	**upper**	**EMM Difference**	**SE[1]**	**DF[2]**	***t***	***p***
	**Systolic Blood Pressure**											
Age group	> 65	Control	136.04	3.28	129	129.55	142.52	-6.74	3.94	129	-1.71	0.089
	> 65	Intervention	129.30	2.72	129	123.91	134.68					
	≤ 65	Control	139.89	1.00	129	137.92	141.87	-8.60	1.41	129	-6.09	0.000
	≤ 65	Intervention	131.30	1.02	129	129.29	133.31					
Gender	female	Control	136.89	1.93	129	133.08	140.70	-7.75	2.36	129	-3.29	0.001
	female	Intervention	129.14	1.61	129	125.95	132.33					
	male	Control	139.04	1.93	129	135.22	142.87	-7.59	2.56	129	-2.96	0.004
	male	Intervention	131.46	1.76	129	127.96	134.95					
	**Weight change (%)**											
Age group	> 65	Control	1.04	1.33	130	-1.60	3.68	-5.25	1.64	130	-3.21	0.002
	> 65	Intervention	-4.21	1.13	130	-6.45	-1.96					
	≤ 65	Control	-0.71	0.41	130	-1.53	0.11	-2.53	0.61	130	-4.19	0.000
	≤ 65	Intervention	-3.24	0.45	130	-4.13	-2.36					
Gender	female	Control	-0.31	0.78	130	-1.85	1.22	-4.05	0.98	130	-4.13	0.000
	female	Intervention	-4.37	0.69	130	-5.72	-3.01					
	male	Control	0.65	0.80	130	-0.93	2.23	-3.73	1.07	130	-3.47	0.001
	male	Intervention	-3.08	0.74	130	-4.55	-1.61					
	**Diastolic Blood Pressure**											
Age group	> 65	Control	84.10	2.40	129	79.36	88.84	-4.32	2.96	129	-1.46	0.147
	> 65	Intervention	79.78	2.03	129	75.77	83.80					
	≤ 65	Control	86.27	0.73	129	84.82	87.72	-5.04	1.03	129	-4.91	0.000
	≤ 65	Intervention	81.22	0.75	129	79.75	82.70					
Gender	female	Control	84.88	1.39	129	82.14	87.62	-5.37	1.74	129	-3.09	0.002
	female	Intervention	79.51	1.19	129	77.16	81.87					
	male	Control	85.49	1.42	129	82.67	88.30	-3.99	1.92	129	-2.08	0.039
	male	Intervention	81.49	1.31	129	78.90	84.08					
	**Quality of Life (SF-8) change**											
Age group	> 65	Control	-1.45	3.56	129	-8.48	5.59	8.33	4.34	129	1.92	0.057
	> 65	Intervention	6.88	2.96	129	1.03	12.74					
	≤ 65	Control	1.23	1.09	129	-0.92	3.39	5.56	1.57	129	3.54	0.001
	≤ 65	Intervention	6.80	1.17	129	4.49	9.10					
Gender	female	Control	1.48	2.09	129	-2.65	5.60	5.21	2.59	129	2.01	0.046
	female	Intervention	6.68	1.78	129	3.16	10.20					
	male	Control	-1.69	2.11	129	-5.86	2.48	8.69	2.85	129	3.05	0.003
	male	Intervention	7.00	2.02	129	3.00	11.00					
	**Food Literacy (SFLQ) change**											
Age group	> 65	Control	2.53	1.74	129	-0.91	5.97	4.07	2.15	129	1.89	0.061
	> 65	Intervention	6.60	1.49	129	3.65	9.54					
	≤ 65	Control	1.84	0.55	129	0.76	2.92	4.79	0.77	129	6.19	0.000
	≤ 65	Intervention	6.64	0.57	129	5.51	7.76					
Gender	female	Control	2.31	1.02	129	0.30	4.33	4.73	1.28	129	3.70	0.000
	female	Intervention	7.04	0.87	129	5.31	8.77					
	male	Control	2.06	1.04	129	0.00	4.12	4.13	1.43	129	2.88	0.005
	male	Intervention	6.19	1.01	129	4.20	8.19					

[1]Standard Error
[2]Degrees of Freedom

Table S10. SF-8 component score summary

	Intervention					Control				
Timepoint	Mean	SD	Q25	Medi an	Q75	Mean	SD	Q25	Medi an	Q75
Overall Health										
Baseline	39.7	10.6	33.2	39.5	49.3	39.3	9.7	33.2	43.3	43.3
Week 12	47.2	10.4	43.3	49.7	55.3	41.1	8.9	33.2	43.3	44.7
Mental Health										
Baseline	41.6	12.9	30.8	43.9	52.6	40.7	11.6	30.8	41.7	49.4
Week 12	46.3	12.5	39.5	50.4	56.9	41.2	11.8	30.8	43.9	52.5
Physical Health										
Baseline	40.6	11.1	30.7	39.9	50.4	41.0	10.9	30.7	40.4	48.9
Week 12	46.1	11.6	39.0	50.4	56.1	42.5	10.6	32.4	44.3	51.2

Table S11. Adherence to app intervention.

	Active users	
Week	n	(%)
1	71	100.0%
2	70	98.6%
3	70	98.6%
4	70	98.6%
5	69	97.2%
6	68	95.8%
7	68	95.8%
8	69	97.2%
9	69	97.2%
10	68	95.8%
11	68	95.8%
12	69	97.2%

Active user is defined as at least one app interaction per week.

Table S12. CONSORT 2025[5] checklist

Section/topic	No	CONSORT 2025 checklist item description	Reported in section
**Title and abstract**			
Title and structured abstract	1a	Identification as a randomised trial	**Title and abstract**
	1b	Structured summary of the trial design, methods, results, and conclusions	**Title and abstract**
**Open science**			
Trial registration	2	Name of trial registry, identifying number (with URL) and date of registration	**Methods**
Protocol and statistical analysis plan	3	Where the trial protocol and statistical analysis plan can be accessed	**.**
Data sharing	4	Where and how the individual de-identified participant data (including data dictionary), statistical code and any other materials can be accessed	**Data availability statement**
Funding and conflicts of interest	5a	Sources of funding and other support (eg, supply of drugs), and role of funders in the design, conduct, analysis and reporting of the trial	**Funding/Methods**
	5b	Financial and other conflicts of interest of the manuscript authors	**Methods**
**Introduction**			
Background and rationale	6	Scientific background and rationale	**Introduction**
Objectives	7	Specific objectives related to benefits and harms	**Introduction**
**Methods**			
Patient and public involvement	8	Details of patient or public involvement in the design, conduct and reporting of the trial	**Methods**
Trial design	9	Description of trial design including type of trial (eg, parallel group, crossover), allocation ratio, and framework (eg, superiority, equivalence, non-inferiority, exploratory)	**Methods**
Changes to trial protocol	10	Important changes to the trial after it commenced including any outcomes or analyses that were not prespecified, with reason	**Methods**
Trial setting	11	Settings (eg, community, hospital) and locations (eg, countries, sites) where the trial was conducted	**Methods**
Eligibility criteria	12a	Eligibility criteria for participants	**Methods +Supplement**

	12b	If applicable, eligibility criteria for sites and for individuals delivering the interventions (eg, surgeons, physiotherapists)	**n.a.**
Intervention and comparator	13	Intervention and comparator with sufficient details to allow replication. If relevant, where additional materials describing the intervention and comparator (eg, intervention manual) can be accessed	**Methods +Supplement**
Outcomes	14	Prespecified primary and secondary outcomes, including the specific measurement variable (eg, systolic blood pressure), analysis metric (eg, change from baseline, final value, time to event), method of aggregation (eg, median, proportion), and time point for each outcome	**Methods +Supplement**
Harms	15	How harms were defined and assessed (eg, systematically, non-systematically)	**Methods**
Sample size	16a	How sample size was determined, including all assumptions supporting the sample size calculation	**Methods**
	16b	Explanation of any interim analyses and stopping guidelines	**n.a.**
Randomisation:			
Sequence generation	17a	Who generated the random allocation sequence and the method used	**Methods**
	17b	Type of randomisation and details of any restriction (eg, stratification, blocking and block size)	**Methods**
Allocation concealment mechanism	18	Mechanism used to implement the random allocation sequence (eg, central computer/telephone; sequentially numbered, opaque, sealed containers), describing any steps to conceal the sequence until interventions were assigned	**Methods**
Implementation	19	Whether the personnel who enrolled and those who assigned participants to the interventions had access to the random allocation sequence	**Methods**
Blinding	20a	Who was blinded after assignment to interventions (eg, participants, care providers, outcome assessors, data analysts)	**Methods**
	20b	If blinded, how blinding was achieved and description of the similarity of interventions	**Methods**
Statistical methods	21a	Statistical methods used to compare groups for primary and secondary outcomes, including harms	**Methods**
	21b	Definition of who is included in each analysis (eg, all randomised participants), and in which group	**Methods**
	21c	How missing data were handled in the analysis	**Methods**
	21d	Methods for any additional analyses (eg, subgroup and sensitivity analyses), distinguishing prespecified from post hoc	**Methods**
**Results**			
Participant flow, including flow diagram	22a	For each group, the numbers of participants who were randomly assigned, received intended intervention, and were analysed for the primary outcome	**Fig. 1**
	22b	For each group, losses and exclusions after randomisation, together with reasons	**Fig. 1**
Recruitment	23a	Dates defining the periods of recruitment and follow-up for outcomes of benefits and harms	**Results**
	23b	If relevant, why the trial ended or was stopped	**n.a.**
Intervention and comparator delivery	24a	Intervention and comparator as they were actually administered (eg, where appropriate, who delivered the intervention/comparator, how participants adhered, whether they were delivered as intended (fidelity))	**Methods**
	24b	Concomitant care received during the trial for each group	**Methods**

Baseline data	25	A table showing baseline demographic and clinical characteristics for each group	**Table 1**
Numbers analysed, outcomes and estimation	26	For each primary and secondary outcome, by group:<br>● the number of participants included in the analysis<br>● the number of participants with available data at the outcome time point<br>● result for each group, and the estimated effect size and its precision (such as 95% confidence interval)<br>● for binary outcomes, presentation of both absolute and relative effect size	**Table 2**<br>**Table S6**
Harms	27	All harms or unintended events in each group	**Results**
Ancillary analyses	28	Any other analyses performed, including subgroup and sensitivity analyses, distinguishing pre-specified from post hoc	**Methods**
**Discussion**			
Interpretation	29	Interpretation consistent with results, balancing benefits and harms, and considering other relevant evidence	**Discussion**
Limitations	30	Trial limitations, addressing sources of potential bias, imprecision, generalisability, and, if relevant, multiplicity of analyses	**Discussion**

Table S13. Study synopsis

1	Title of Study	Randomised, controlled trial to evaluate the effectiveness of Oviva Direkt in reducing blood pressure in patients with hypertension (HYPE STUDY)
2	Medical Condition	Essential (primary) hypertension (ICD-10-GM, I10)
3	Hypothesis	Empirical: Oviva Direkt Hypertension, in addition to standard of care, improves systolic blood pressure after 12 weeks in patients with hypertension.<br><br>Statistical: Patients using Oviva Direkt Hypertension in addition to standard of care will show a smaller mean systolic blood pressure after 12 weeks of treatment compared with patients using standard of care only for 12 weeks.
4	Key Inclusion Criteria	Essential Hypertension (ICD-10-GM, I10)<br>Age 18-75 years<br>Sex female and male
5	Key Exclusion Criteria	Current or recent participation (within the past 12 months) in a digital lifestyle intervention or clinical investigation targeting blood pressure and/or body weight reduction<br>Weight loss >5% in the past 6 months (intentional or unintentional)<br>Living in a household with another study participant for a digital lifestyle intervention
6	Trial type	Interventional randomised controlled evaluator-blinded study
7	Intervention	Experimental group: Oviva Direkt Hypertension (App-based digital lifestyle intervention) + standard of care<br>Control group: standard of care<br>Duration of intervention per patient: 12 weeks<br>Follow up per patient: 4 assessments (i.e., at baseline, 4, 8, and 12 weeks)
8	Key Procedures	Patient screening<br>Patient enrolment<br>Intervention delivery<br>Outcome assessment
9	Outcomes	Primary endpoint:<br>Mean systolic blood pressure measured by home-based blood pressure monitoring at week 12<br><br>Key secondary endpoint(s):<br>Percent change in body weight between baseline and 12 weeks measured by home-based monitoring<br>Mean diastolic blood pressure measured by home-based blood pressure monitoring at week 12<br>Change in health-related quality of life between baseline and 12 weeks measured by SF-8 questionnaire<br>Change in patient food literacy between baseline and 12 weeks

		measured by SFLQ questionnaire<br><br>Assessment of safety:<br>Collection, recording and reporting of adverse events to the sponsor will be completed according to the sponsor's SOP for the Recording, Management and Reporting of Adverse Events (AE). Safety variables include adverse events and adverse device effects. Adverse events are summarised overall, by severity, and by relationship to the medical device.
10	Statistical Analysis	Intention to treat analysis of covariance (ANCOVA) for primary and secondary endpoints to compare intervention and control group.<br>Data imputation uses multiple imputations by chained equations (MICE).<br>Subgroup analyses focus on differential effects for age groups and gender.<br>The expected number of participants to be assigned to the trial is 134. This number accounts for an expected 25% dropout rate, resulting in 100 complete cases.
11	Sample Size	to be assessed for eligibility: n = 400<br>to be assigned to the trial: n = 134<br>to be analysed: n = 134 (intention-to-treat)
12	Trial Duration	recruitment period (months): 3<br>first participant in to last participant out (months): 6
13	Discussion Expected Risks and Benefits	Participating in this trial may provide several benefits to individual participants. These benefits include potential improvements in blood pressure control, body weight reduction, and health-related quality of life. By using Oviva Direkt Hypertension, participants will have access to a well-established and guideline-endorsed lifestyle guidance program delivered through a technology based on proven behaviour change theories. The intervention aims to support participants in developing healthy nutritional habits and increasing physical activity to reduce blood pressure and lose body weight. There are no known risks resulting in direct harm in using Oviva Direkt Hypertension.

References

1. McEvoy JW, McCarthy CP, Bruno RM, Brouwers S, Canavan MD, Ceconi C, Christodorescu RM, Daskalopoulou SS, Ferro CJ, Gerdts E, Hanssen H, Harris J, Lauder L, McManus RJ, Molloy GJ, Rahimi K, Regitz-Zagrosek V, Rossi GP, Sandset EC, Scheenaerts B, Staessen JA, Uchmanowicz I, Volterrani M, Touyz RM, ESC Scientific Document Group, Abreu A, Olsen MH, Ambrosetti M, Androulakis E, Bang LE, Bech JN, Borger MA, Boutouyrie P, Bronze L, Buccheri S, Dalmau R, De Pablo Zarzosa MC, Delles C, Fiuza MM, Gabulova R, Haugen BO, Heiss C, Ibanez B, James S, Kapil V, Kayikçioglu M, Køber L, Koskinas KC, Locati ET, MacDonald S, Mihailidou AS, Mihaylova B, Mindham R, Mortensen MB, Nardai S, Neubeck L, Nielsen JC, Nilsson PM, Pasquet AA, Pedro MM, Prescott E, Rakisheva A, Rietzschel E, Rocca B, Rossello X, Schmid J-P, Shantsila E, Sudano I, Timóteo AT, Tsivgoulis G, Ungar A, Vaartjes I, Visseren F, Voeller H, Vrints C, Witkowski A, Zennaro M-C, Zeppenfeld K, Shuka N, Laredj N, Pavo N, Mirzoyev U, Van De Borne P, Sokolović Š, Postadzhiyan A, Samardzic J, Agathangelou P, Widimsky J, Olsen MH, El-Kilany WM, Pauklin P, Laukkanen JA, Boulestreau R, Tsinamdzgvrishvili B, Kintscher U, Marketou M, Páll D, Hrafnkelsdóttir ÞJ, Dolan E, Wolak T, Bilo G, Tundybayeva MK, Mirrakhimov E, Trusinskis K, Kiwan G, Msalem O, Badarienė J, Banu C-A, Balbi MM, Caraus A, Boskovic A, Mouine N, Vromen T, Bosevski M, Midtbø HB, Doroszko A, Dores H, Badila E, Bini R, Simić DV, Fras Z, Mazón P, Spaak J, Burkard T, Barakat E, Abdessalem S, Gunes Y, Sirenko YM, Brady AJB, Khamidullaeva GA. 2024 ESC Guidelines for the management of elevated blood pressure and hypertension. *European Heart Journal* 2024;**45**:3912–4018.

2. Mancia G, Kreutz R, Brunström M, Burnier M, Grassi G, Januszewicz A, Muiesan ML, Tsioufis K, Agabiti-Rosei E, Algharably EAE, Azizi M, Benetos A, Borghi C, Hitij JB, Cifkova R, Coca A, Cornelissen V, Cruickshank JK, Cunha PG, Danser AHJ, Pinho RMD, Delles C, Dominiczak AF, Dorobantu M, Doumas M, Fernández-Alfonso MS, Halimi J-M, Járai Z, Jelaković B, Jordan J, Kuznetsova T, Laurent S, Lovic D, Lurbe E, Mahfoud F, Manolis A, Miglinas M, Narkiewicz K, Niiranen T, Palatini P, Parati G, Pathak A, Persu A, Polonia J, Redon J, Sarafidis P, Schmieder R, Spronck B, Stabouli S, Stergiou G, Taddei S, Thomopoulos C, Tomaszewski M, Van De Borne P, Wanner C, Weber T, Williams B, Zhang Z-Y, Kjeldsen SE. 2023 ESH Guidelines for the management of arterial hypertension The Task Force for the management of arterial hypertension of the European Society of Hypertension: Endorsed by the International Society of Hypertension (ISH) and the European Renal Association (ERA). *Journal of Hypertension* 2023;**41**:1874–2071.

3. Wirtz MA, Schulz A, Brähler E. Confirmatory and bi-factor analysis of the Short Form Health Survey 8 (SF-8) scale structure in a German general population sample. *Health Qual Life Outcomes* 2021;**19**:73.

4. Gréa Krause C, Beer-Borst S, Sommerhalder K, Hayoz S, Abel T. A short food literacy questionnaire (SFLQ) for adults: Findings from a Swiss validation study. *Appetite* 2018;**120**:275–280.

5. Hopewell S, Chan A-W, Collins GS, Hróbjartsson A, Moher D, Schulz KF, Tunn R, Aggarwal R, Berkwits M, Berlin JA, Bhandari N, Butcher NJ, Campbell MK, Chidebe RCW, Elbourne D, Farmer A, Fergusson DA, Golub RM, Goodman SN, Hoffmann TC, Ioannidis JPA, Kahan BC, Knowles RL, Lamb SE, Lewis S, Loder E, Offringa M, Ravaud P, Richards DP, Rockhold FW, Schriger DL, Siegfried NL, Staniszewska S, Taylor RS, Thabane L, Torgerson D,

Vohra S, White IR, Boutron I. CONSORT 2025 statement: updated guideline for reporting randomised trials. *BMJ* 2025:e081123.
